# Evaluating the health and economic impact of osteoarthritis pain in the workforce: results from the National Health and Wellness Survey

**DOI:** 10.1186/1471-2474-12-83

**Published:** 2011-04-28

**Authors:** Marco daCosta DiBonaventura, Shaloo Gupta, Margaret McDonald, Alesia Sadosky

**Affiliations:** 1 Kantar Health, 11 Madison Ave., New York, NY 10010, USA; 2 Pfizer, Inc., 235 East 42 Street, New York, NY, 10017, USA

## Abstract

**Background:**

There has been increasing recognition that osteoarthritis (OA) affects younger individuals who are still participants in the workforce, but there are only limited data on the contribution of OA pain to work productivity and other outcomes in an employed population. This study evaluated the impact of OA pain on healthcare resource utilization, productivity and costs in employed individuals.

**Methods:**

Data were derived from the 2009 National Health and Wellness Survey. Univariable and multivariable analyses were used to characterize employed individuals (full-time, part-time, or self-employed) ≥20 years of age who were diagnosed with OA and had arthritis pain in the past month relative to employed individuals not diagnosed with OA or not experiencing arthritis pain in the past month. Work productivity was assessed using the Work Productivity and Activity Impairment (WPAI) questionnaire; health status was assessed using the physical (PCS) and mental component summary (MCS) scores from the SF-12v2 Health Survey and SF-6D health utilities; and healthcare utilization was evaluated by type and number of resources within the past 6 months. Direct and indirect costs were estimated and compared between the two cohorts.

**Results:**

Individuals with OA pain were less likely to be employed. Relative to workers without OA pain (n = 37,599), the OA pain cohort (n = 2,173) was significantly older (mean age 52.1 ± 11.5 years vs 41.4 ± 13.2 years; *P *< 0.0001) and with a greater proportion of females (58.2% vs 45.9%; *P *< 0.0001). OA pain resulted in greater work impairment than among workers without OA pain (34.4% versus 17.8%; *P *< 0.0001), and was primarily due to presenteeism (impaired activity while at work). Health status, assessed both by the SF-12v2 and the SF-6D was significantly poorer among workers with OA pain (*P *< 0.0001), and healthcare resource utilization was significantly higher (*P *< 0.0001) than workers without OA pain. Total costs were higher in the OA pain cohort ($15,047 versus $8,175; *P *< 0.0001), driven by indirect costs that accounted for approximately 75% of total costs.

**Conclusions:**

A substantial proportion of workers suffer from OA pain. After controlling for confounders, the impact of OA pain was significant, resulting in lower productivity and higher costs.

## Background

Osteoarthritis (OA) is a degenerative joint disease that affects almost 27 million adults in the United States (US) and is ranked among the top three causes of disability [1,2]. Pathologic features of OA include loss of articular cartilage and concomitant development of osteophytes at the joint margins. In the clinical setting, pain is a major complaint among patients with OA that also contributes to patient report of reduced joint function. In line with this, management strategies of OA include reduction of pain and improvement of function as the main targets [3-7].

OA has been considered an age-related disease, and substantial functional impairment, decreased quality of life, and increased healthcare resource utilization have been reported among older patients with OA [8-12]. However, there is increasing recognition of the effects of OA on younger patients who are still participants in the workforce, and several studies have documented that OA is associated with substantial reductions in productivity among employed individuals [13-15].

The importance of lost productivity in arthritis has recently been highlighted by the endorsement by OMERACT (Outcome Measures in Rheumatology) of work as an outcome, although no particular measure was recommended for its evaluation [16]. While it has also been suggested that indirect costs related to lost work productivity are the primary driver of the economic burden of OA [17-19], some studies reported indirect costs that were lower than direct costs [20-22]. Since there are no standardized methods for estimating indirect costs in OA [23], these contrasting results are likely due to differences in populations and methodologies, including inadequately accounting for the contribution of presenteeism (reduced productivity while at work) to indirect costs. In fact, presenteeism has been suggested to be the primary source of lost productive time and indirect costs among workers with chronic pain conditions including arthritis [24]. This was recently demonstrated in a study reporting a considerably increased risk of presenteeism among workers with arthritis relative to individuals without chronic conditions (odds ratio: 8), whereas the risk of absenteeism was not increased [25].

In workers with OA, reductions in work productivity, including presenteeism, appear to be associated with increased patient-reported OA severity [26], yet data on the contribution of OA pain to work productivity and other outcomes in an employed population are limited. A study by White et al. [21] estimated the economic burden of patients with OA who were receiving pain pharmacotherapy, but the relative impact of pain on costs was not addressed. A more direct effect of OA pain was demonstrated in an analysis of data from the Longitudinal Examination of Arthritis Pain (LEAP) study [27]. Results from LEAP suggested that weekly fluctuations in OA pain were associated with changes in levels of daily activities/functioning, work absenteeism, sleep interference, and healthcare resource use. In an analysis of four measures of presenteeism (the Health and Labor Questionnaire [HLQ]; the Work Limitations Questionnaire [WLQ]; the World Health Organization's Health and Work Performance Questionnaire [HPQ]; and the Work Productivity and Activity Impairment Questionnaire [WPAI]), Zhang et al. [28] observed a significant association between pain and the risk of presenteeism, but only weak associations between pain severity and hours lost. Another study implicated acute pain exacerbations as a factor in lost productivity in a population of employed adults with arthritis, however, the type of arthritis was not specified [13]. In that study, the magnitude of lost productivity was mediated by the proportion of workers who lost work time rather than the amount of time lost. This use of proportions rather than amount of time was further considered in a combined OA and rheumatoid arthritis population as part of a validation study comparing responsiveness of five at-work productivity measures: the Workplace Activity Limitations Scale (WALS); the 6-item Stanford Presenteeism Scale [SPS-6]; the Endicott Work Productivity Scale [EWPS]; the Rheumatoid Arthritis Work Instability Scale [WIS]; and the Work Limitations Questionnaire [WLQ]) [29]. As indicated by the mean score on each of the measures, only mild at-work productivity losses were reported, and the correlations between pain severity in the past week and scores on the five measures were only moderate.

To increase our knowledge of the consequences of OA pain in the workforce, the objective of this study was to use the National Health and Wellness Survey (NHWS) to evaluate the impact of OA pain on direct and indirect costs and health provider visits, as well as on measures of general health status in an employed population. The NHWS is a cross-sectional, self-administered, internet-based questionnaire administered annually to a nationwide sample of adults (aged 18 or older).

## Methods

### Data source and population

Data were derived from the 2009 National Health and Wellness Survey (NHWS) that included information on 75,000 individuals in the United States (US). To represent the demographic composition of the US adult population, the data are weighted by gender, age and race/ethnicity http://www.chsinternational.com/nhws.html.

This analysis was performed using data only for those respondents who were ≥ 20 years of age and were currently employed full-time, part-time, or self-employed. Subjects meeting these criteria were stratified into two cohorts. The first cohort was defined as workers who self-reported that they have been diagnosed with OA and were experiencing arthritis pain in the past month, and the comparator cohort consisted of workers who self-reported that they were *either *not diagnosed with osteoarthritis *or *not experiencing arthritis pain in the past month. Self-report of an OA diagnosis was based on two questions. The first question determined whether the subject had been diagnosed with arthritis by a physician (response of yes/no), and the second question elicited the type of arthritis that the subject has, with potential responses of osteoarthritis, rheumatoid arthritis, psoriatic arthritis, and not sure. No clinical information (e.g., X-ray findings) was asked of participants to define the presence of OA.

### Outcomes evaluated

The demographic and health characteristics of the two cohorts were characterized and compared. These characteristics included age, gender, race/ethnicity, marital status, education, income, insurance status, employment type, body mass index (BMI), and Charlson Comorbidity Index (CCI) [30].

Work productivity was assessed using the Work Productivity and Activity Impairment (WPAI) questionnaire [31]. The WPAI is a self-reported questionnaire consisting of four subscales that evaluate absenteeism, presenteeism, overall work impairment, and activity impairment during the previous seven days, generated in the form of percentages, with higher values indicating greater impairment. The WPAI used in the current analysis was not specific for OA.

Health-related quality of life was assessed using the physical (PCS) and mental component summary (MCS) scores from the self-reported SF-12v2 Health Survey [32]. Scores for the PCS and MCS are normed to the US population (mean = 50, SD = 10) and vary from 0 to 100, with higher scores indicating better quality of life. Additionally, health utility scores for both cohorts were calculated using the SF-6D [33], which provides a preference-based single index measure for health status and varies from 0.29 to 1.

Healthcare utilization, regardless of cause, was evaluated by type and number of resources that the patients reported using within the past six months. These resources included number of prescriptions and provider visits for both traditional healthcare (physician, emergency room [ER], and hospitalizations) and non-traditional healthcare e.g., acupuncturist, herbalist, etc.

The direct medical costs evaluated in this analysis included only traditional provider visits (i.e. physician, ER, and hospitalizations). These costs were estimated by multiplying the units of resource categories for six months by the average cost of the resource derived from the Medical Expenditure Panel Survey database [34-36], and then multiplying by two to project annual costs. Indirect costs associated with lost productivity for any reason were calculated among both cohorts using the method of Lofland et al. [37] based on data from the WPAI and median annual income values obtained through the Bureau of Labor Statistics (BLS) [38]. For each respondent, the percent overall work impairment (obtained from the WPAI) was multiplied by the annual income. Direct and indirect costs were summed to provide an estimate of total costs.

### Statistical analyses

Univariable analysis was used to examine differences in demographic and clinical characteristics between the two groups. Analysis of quality of life and work productivity were performed using multivariable models with the following demographic and clinical characteristics as covariates: age range (coded as 20-39 vs. 40-64 and ≥ 65 years), gender, race/ethnicity (coded as non-Hispanic white, non-Hispanic black, Hispanic, or other), education (more than high school vs. high school equivalent degree or less), income (< $25 K, $25 K to < $50 K, $50 K to < $75 K, ≥ $75 K, or decline to answer), CCI (0 vs. ≥ 1), health insurance (yes vs. no), BMI (underweight, normal, overweight, obese, or decline to answer), employment (full-time, part-time, or self-employed), traditional healthcare visits (yes vs. no), non-traditional healthcare visits (yes vs. no), prescription drug use (yes vs. no), ER visits (yes vs. no), and hospitalization (yes vs. no).

Determination and analysis of costs were not adjusted for covariates. Generalized linear models (GLMs) were conducted to estimate work productivity and activity impairment using a negative binomial distribution to account for skewness and provide best fit. Significant differences between the two groups were examined using Wald chi-square tests for categorical outcomes and independent-samples t-tests for continuous outcomes. For all analyses, a *P *value < 0.05 was considered statistically significant.

## Results

Of the 39,772 workers who met the inclusion criteria, 2,173 were diagnosed with OA and experienced arthritis pain in the past month, and thus were included in the OA pain cohort. The peripheral joints (ankles, elbows, feet, fingers, hands, hips, knees, neck, shoulders, wrists, other) were affected in 98.1% of these patients, and the spine was affected in 43.7%. The comparator cohort consisted of workers not diagnosed with osteoarthritis or not experiencing arthritis pain in the past month (n = 37,599), with 10.7% and 2.5% reporting peripheral and spinal involvement, respectively.

Weighted univariable analysis of demographic characteristics showed that workers in the OA pain cohort were not only significantly older than the comparator group (mean 52.1 ± 11.5 vs 41.4 ± 13.2 years; *P *< 0.0001), but also had a greater comorbidity burden as indicated by the CCI (mean 0.8 ± 1.2 vs 0.3 ± 0.9; *P *< 0.0001). Additionally, workers in the OA pain cohort tended toward obesity (BMI ≥ 30); mean BMI 31.0 ± 7.4 vs 28.2 ± 6.7 (*P *< 0.0001). As shown in Table 1, there was a significantly greater proportion of females in the OA pain cohort (58.2 vs 45.9%; *P *< 0.0001), and while both cohorts were predominantly non-Hispanic and White, this demographic had greater representation in the OA pain group. Significantly lower proportion of workers with OA pain reported full-time employment and an income ≥ $75,000; no differences were observed between cohorts in level of education.

**Table 1:** Weighted univariable statistics (to reflect the US population) for demographic characteristics of workers with OA pain compared with workers without OA pain.

Variable	OA Pain (n = 2,173)		Without OA pain (n = 37,599)		*P*
	
	n	Weighted percent (SE)	n	Weighted percent (SE)	
Age range					
20-39 years	267	14.5 ± 0.8	16,123	47.7 ± 0.3	< 0.0001
40-64 years	1,453	71.8 ± 1.1	18,571	47.8 ± 0.3	< 0.0001
≥ 65 years	453	13.7 ± 0.9	2,905	4.5 ± 0.1	< 0.0001
Gender					
Male	954	41.8 ± 1.1	19,824	54.1 ± 0.3	< 0.0001
Female	1,219	58.2 ± 1.1	17,775	45.9 ± 0.3	< 0.0001
Race/ethnicity					
White, non-Hispanic	1,792	78.0 ± 1.1	26,347	66.5 ± 0.3	< 0.0001
Black, non-Hispanic	169	8.4 ± 0.8	4,434	12.1 ± 0.2	< 0.0001
Hispanic	99	8.3 ± 0.8	3,569	14.6 ± 0.2	< 0.0001
Other	113	5.3 ± 0.5	3,249	6.8 ± 0.1	0.0032
Education					
High school graduate or less	376	17.9 ± 0.9	6,211	16.9 ± 0.2	0.3053
More than high school	1,797	82.2 ± 0.9	31,387	83.1 ± 0.2	0.3067
Income					
< $25,000	294	13.5 ± 0.8	4,205	11.6 ± 0.2	0.0183
$25,000 to $49,999	667	30.4 ± 1.1	10,807	29.1 ± 0.3	0.2218
$50,000 to $74,999	533	24.8 ± 1.0	9,109	24.2 ± 0.2	0.5271
≥ $75,000	579	26.5 ± 1.1	11,830	30.9 ± 0.3	<.0001
Decline to answer	100	4.8 ± 0.5	1,648	4.2 ± 0.1	0.2813
Employment					
Full time	1,200	57.3 ± 1.2	26,088	70.7 ± 0.3	< 0.0001
Part time	552	23.9 ± 1.1	7,139	18.3 ± 0.2	< 0.0001
Self-employed	421	18.8 ± 0.9	4,372	11.0 ± 0.2	< 0.0001
Health insurance					
Yes	1,880	85.4 ± 0.8	31,143	81.8 ± 0.2	< 0.0001
No	293	14.6 ± 0.8	6,456	18.2 ± 0.2	< 0.0001
BMI					
Underweight	19	0.8 ± 0.2	629	1.7 ± 0.1	< 0.0001
Normal	416	20.1 ± 1.0	11,889	32.1 ± 0.3	< 0.0001
Overweight	660	30.0 ± 1.1	12,679	33.6 ± 0.3	0.0011
Obese	1,044	47.5 ± 1.2	11,737	30.9 ± 0.3	< 0.0001
Decline to answer	34	1.5 ± 0.3	665	1.8 ± 0.1	0.403

**Figure 1:**
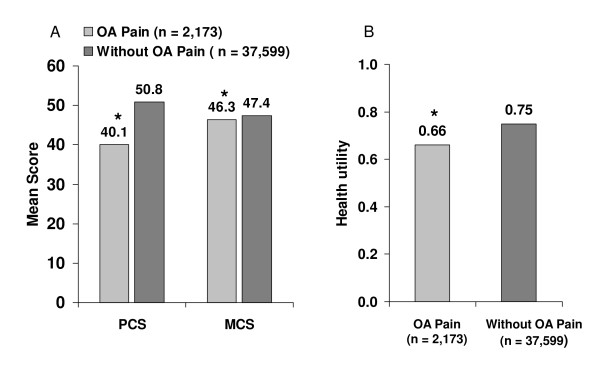
**Health status among individuals with OA pain relative to comparators**. A) Physical Component (PCS) and Mental Component Summary scores on the SF-12v2 (range of 0-100, higher scores indicate better health) adjusted for covariates and normed to the US population (mean = 50, SD = 10). B) Health utility score on the SF-6D (range of 0 = death to 1 = perfect health). *P < 0.0001 versus the comparator cohort.

Health status, as described by the PCS and MCS summary components of the SF-12v2 (Figure 1A) and SF-6D health utility scores adjusted for covariates (Figure 1B) was significantly lower among workers with OA pain (*P *< 0.0001). For the SF-12v2, the difference between cohorts was greater for the PCS. In multivariable analysis after controlling for covariates, these significant differences were maintained, with PCS and MCS scores that were lower in the OA pain cohort by 7.6 (95% CI 8.0, 7.3) and 1.2 (95% CI 1.6, 0.8) points, respectively, and 0.07 points lower (95% CI, 0.08, 0.07) for the SF-6D.

**Figure 2:**
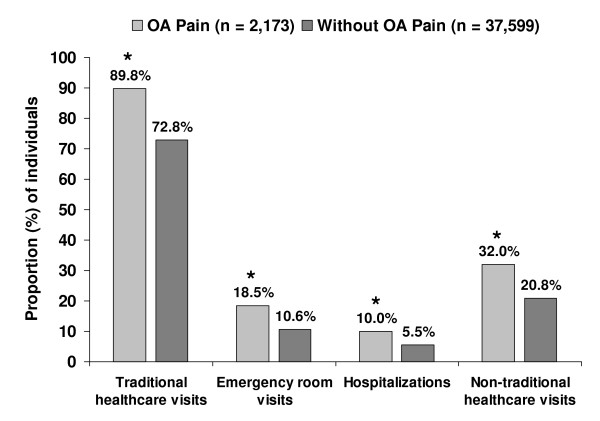
**Adjusted healthcare resource utilization among workers with OA pain relative to workers without OA pain**.

Health resource utilization reported by workers over the previous 6-month period and adjusted for covariates was significantly higher in the OA pain cohort than comparators across provider visit categories including use of non-traditional healthcare (Figure 2). Additionally, workers with OA pain were currently prescribed a mean of 5.3 ± 4.9 medications, more than twice that of workers without OA pain, 2.1 ± 3.4 (*P *< 0.0001).

**Figure 3:**
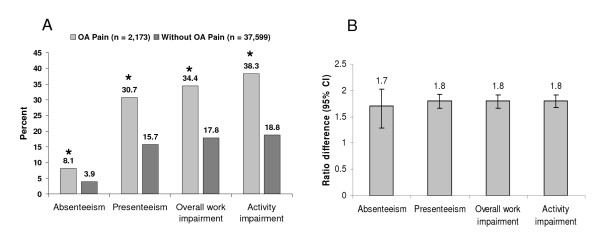
**Work productivity evaluated using the Work Productivity and Impairment (WPAI) questionnaire**. A) Percent work activity impairment. *P < 0.0001 versus controls. B) Impact of impairment; the ratio difference indicates the magnitude of impairment for workers with OA pain relative to workers without OA pain. All values are adjusted for covariates. *P < 0.0001 for all ratio differences.

**Figure 4:**
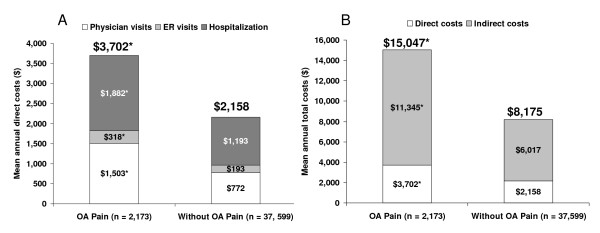
**Unadjusted mean annual costs in the OA pain and comparator cohorts**. A) Direct medical costs for provider visits. B) Total costs including estimated indirect costs plus direct medical costs of provider visits.*P < 0.0001 versus controls.

As shown in Figure 3, adjusted work and activity was impaired to a significantly greater extent in the OA pain cohort relative to the comparator cohort. While in both cohorts, lost productivity due to presenteeism was almost 4-times greater than that due to absenteeism, both percent presenteeism and percent absenteeism were significantly higher among workers in the OA pain cohort (Figure 4A). Workers with OA pain reported losing 31% of productive time while at work and 8% through absenteeism, compared with comparators who lost 16% of productive time while at work and 4% through absences (*P *< 0.0001 for both comparisons). The ratio differences shown in Figure 4B indicate the magnitude of impairment for the OA cohort relative to the comparator group; for all outcomes, lost productivity and impairment was almost twice as high in the OA pain cohort (*P *< 0.0001 for all ratio differences). In terms of actual hours, workers with OA pain lost a mean of 2.7 ± 7.1 hours during the past week due to absenteeism, and a mean of 9.7 ± 9.7 hours due to presenteeism. In contrast, the comparator cohort lost 1.4 ± 5.6 and 5.2 ± 8.6 hours to absenteeism and presenteeism, respectively; *P *< 0.0001 for both comparisons with the OA pain cohort.

All presented costs represent actual costs unadjusted for covariates. Direct medical costs were significantly higher in the OA pain cohort across all provider categories (Figure 4A), resulting in total direct medical costs that were more than 1.5-times higher than for the comparator group, $3,702 vs $2,158 (*P *< 0.0001). In both cohorts, hospitalization costs accounted for slightly more than half of the direct costs, 51% and 55% for OA pain and the comparator, respectively. Total costs were almost twice as high among workers with OA pain cohort as in workers without OA pain, $15,047 and $8,175, respectively (*P *< 0.0001), and were primarily driven by indirect costs resulting from lost productivity.

## Discussion

This study not only confirms the substantial presence and economic impact of OA in the workforce that has been suggested by other studies [14,15,21], but supports other evidence that OA pain is a contributory factor to the economic and individual worker burden. Notably, among the employed population that served as the basis for this study, the prevalence of workers with OA who had arthritis pain during the past month was greater than 5%. Although pain is primary symptom of OA, it is likely that the comparator cohort contained a proportion of workers with OA who did not have pain in the past month, suggesting that the overall presence of OA in the workforce is likely to be higher.

While the demographics of the OA pain cohort relative to the comparator were generally consistent with what might be expected for OA, i.e. older and female, the mean age of the OA pain cohort (52 years) nevertheless demonstrates the presence of OA in a younger demographic having a potential for additional years of workforce participation.

Workers with OA pain reported significantly lower health status than the comparator cohort even after adjusting for relevant covariates. Not surprisingly, since OA and pain affects physical functioning, there was a greater effect on physical components of health status, although the difference in mental components was also significant. The health utility score was also significantly lower among the workers with OA pain relative to the comparators; health utility scores are important from the economic perspective since they are frequently incorporated into economic analyses. The adjusted difference between cohorts on the SF-12v2 PCS score (7.6 points) and the SF-6D health utility score (0.07 points) exceeded the values that are considered clinically meaningful; > 3 and 0.03 points, respectively [39,40].

The data also show that workers with OA pain had more health provider visits across categories and were prescribed twice as many medications than the comparator cohort, although whether these visits or medications were specifically related to pain could not be ascertained. It is also interesting to note that non-traditional health resources were used by a greater proportion of individuals with OA pain, since these visits may not necessarily be covered by insurance plans.

Workers with OA pain were characterized by significantly greater work and activity impairment relative to comparators. In the OA pain cohort overall, approximately one-third (34%) of worker productivity was lost. Presenteeism has been a neglected component of assessing lost productivity, and is an outcome that can best be captured by studies that are based on patient report. As far as we can ascertain, this is the first study in OA that evaluated presenteeism in a complete context of worker impairment along with absenteeism. Consistent with the results suggested by the literature on the general arthritis population [13,24,25,41], presenteeism was the primary source of work impairment, with almost 10 hours per week lost while at work; almost 4-fold greater than for absenteeism.

A number of measures of productivity are available that include assessment of presenteeism, and a recent study has shown variability in the ability of four of these measures, including the WPAI, to estimate presenteeism [28]. However, among the four measures, the WPAI was the only one having a complete response rate, possibly related to its brevity and ease of use; several of the other measures are associated with an administration burden because of their length. It is for this reason that the WPAI is among the most commonly used productivity measures, and was considered appropriate for the current study. Additionally, the significant association between pain and presenteeism observed in that study further suggests that the high burden of lost productivity observed in our OA pain cohort is due at least in part to the pain component [28].

Mean annual unadjusted costs in the OA pain cohort, which were significantly higher than the comparator cohort, reflected the substantial impact of lost productivity, with estimated indirect costs of $11,345. These indirect costs account for approximately 75% of total costs, and are almost twice as high as the indirect costs of the comparator cohort ($6,017). Interestingly, among workers without OA pain, indirect costs accounted for a similar proportion of total costs, suggesting the general importance of lost productivity in economic evaluations of health and disease. For direct costs, although only 10% of the OA pain cohort reported hospitalizations, this resource category was the primary cost driver, most likely due to the high cost per event.

All outcomes, except for costs, were adjusted for relevant covariates. Since many factors (such as age, ethnicity, gender, socioeconomic status, and insurance) are likely to impact health care seeking behavior by patients and management strategies by providers, caution should be applied when interpreting the implications of the above cost analysis. Future cost analyses controlling for such covariates may yield slightly different estimates.

### Limitations

Although strengths of this study that may enhance its generalizability include a large sample size and population-level analysis based on a weighted assessment to reflect the demographic composition of the US population, there are certain limitations that need to be considered. An important limitation is that the data used in the analyses are based on patient self-report, without clinical verification of an OA diagnosis, and thus are subject to the biases that are inherent in this type of data presentation. It is possible that some patients reported that they were diagnosed with OA when, in fact, they were not. Conversely, some patients may have thought they were diagnosed with another condition when, in fact, it was OA. As a result, additional error could have been introduced into the analyses. Nevertheless, as previously noted, patient report provides an important source for evaluating outcomes, and is the primary means of determining presenteeism.

Since this was part of a large general health survey, the version of the WPAI that was used was not OA specific. Therefore, the observed relationships between OA pain and productivity should be considered associative rather than causal, since direct causality cannot be inferred regarding the OA pain as a source of lost productivity, especially given that workers with OA pain were characterized by a significantly greater comorbidity burden than workers without OA pain. Similarly, the higher resource utilization and costs cannot be ascribed specifically to the OA nor the pain, since there are no claims linking resource use with the disease and symptoms of interest. Nevertheless, lost productivity in the OA pain cohort was twice as high as the comparator cohort, with both total costs and indirect costs also almost twice as high, suggesting a substantial burden associated with OA pain that may be of special concern to employers.

Another limitation was that the questionnaire captured neither the type of employment nor the specific site of OA pain. Since these variables are likely to impact productivity as well as management strategies, their absence may reduce the generalizability of the results.

Because the source of our data was not a medical claims database, only the number of prescribed drugs by patient report was captured, and our inability to estimate the costs associated with pharmacotherapy for inclusion in direct costs is another limitation. However, extrapolating from a study that reported total pharmacotherapy costs of $2,941 among patients with OA receiving pain therapy [21], and adding these costs to the direct costs for provider visits estimated in the current study would result in total direct costs that are approximately half of the indirect costs. Our derivation of annual costs was based on extrapolation of 6 month data to 1 year. However, this method may not adequately reflect actual resource utilization over a 12-month period.

## Conclusions

Results from this study highlight the fact that OA pain is not necessarily relegated to an older population, but is present in an under-recognized proportion of the workforce. Despite the above limitations, the data show that presence of OA pain has a profound impact on quality of life, work productivity, and healthcare resource use among workers. Even after controlling for confounding variables, workers with OA pain reported significantly reduced health status and significant work impairment. Notably, this study also demonstrated the importance of presenteeism as a contributing factor to lost productivity. These combined factors resulted in costs that were significantly higher than among workers without OA pain, and were driven by indirect costs, which appeared to primarily result from work impairment that occurred while at work (presenteeism) rather than absence from work. These results not only expand our knowledge of the burden of OA, but they can also inform employers and health care providers on the contribution of OA pain to patient productivity and quality of life. Such information may potentially be of use when considering how to evaluate management strategies for alleviating the worker and employer burdens associated with this condition in the general population.

## Competing interests

Alesia Sadosky and Margaret McDonald are employees and stockholders of Pfizer Inc, the sponsor of this study. Marco DiBonaventura and Shaloo Gupta are employees of Kantar Health, who conducted the National Health and Wellness Survey and analyzed the data on behalf of Pfizer Inc.

## Authors' contributions

All authors contributed to the study design, statistical analysis plan, results interpretation, and review of the draft manuscript; the final manuscript was read and approved by all authors.

## Pre-publication history

The pre-publication history for this paper can be accessed here:

http://www.biomedcentral.com/1471-2474/12/83/prepub
